# Multiple molecular detection of respiratory viruses and associated signs of airway inflammation in racehorses

**DOI:** 10.1186/s12985-016-0657-5

**Published:** 2016-11-29

**Authors:** Nadia Doubli-Bounoua, Eric A. Richard, Albertine Léon, Pierre-Hugues Pitel, Stéphane Pronost, Guillaume Fortier

**Affiliations:** 1 LABÉO Frank Duncombe, Caen, France; 2 Normandie Université, UNICAEN, EA 4655, U2RM, Caen, France

**Keywords:** Equine rhinitis virus -B, qPCR, Tracheal wash, Coughing, Inflammatory airway disease

## Abstract

**Background:**

The potential involvement of viruses in inflammatory airway disease (IAD) was previously investigated through either serology or PCR from nasopharyngeal swabs (NS). The aims of this study were to determine the prevalence and incidence of viral genome detection by qPCR in the equine airways, and their association with respiratory clinical signs.

**Methods:**

Both NS and tracheal washes (TW) were collected monthly on 52 Standardbred racehorses at training, over 27 consecutive months (581 samples). Equid herpesviruses (EHV)-1, −4, −2 and −5, equine rhinitis virus-A and -B (ERBV), equine adenovirus-1 and −2, equine coronavirus and equine influenza virus were systematically investigated in both NS and TW. Nasal discharge, coughing, tracheal mucus score and TW neutrophil proportions were simultaneously recorded.

**Results:**

Genome for 7/10 viruses were detected at least once throughout the study; up to 4 different viruses being also concomitantly detected. Monthly incidence in TW was respectively 27.9% (EHV-5), 24.8% (EHV-2), 7.1% (ERBV), 3.8% (EHV-4), 1.9% (EAdV1) and 0.2% (EHV-1; ERAV). Neither agreement nor correlation between NS and TW was found for respectively genome detection and viral loads. Detection of viral genome in NS was not associated with any clinical sign. Coughing was significantly associated with TW detection of EHV-2 DNA (OR 3.1; *P* = 0.01) and ERBV RNA (OR 5.3; *P* < 0.001). Detection of EHV-2 DNA in TW was also significantly associated with excess tracheal mucus (OR 2.1; *P* = 0.02).

**Conclusions:**

Detection and quantification of EHV-2 and ERBV by qPCR in TW, but not in NS, should be considered when investigating horses with IAD.

**Electronic supplementary material:**

The online version of this article (doi:10.1186/s12985-016-0657-5) contains supplementary material, which is available to authorized users.

## Background

Lower airway troubles and especially inflammatory airway disease (IAD) represent one of the main causes of poor-performance for racehorses [1]. This syndrome, which may affect horses of any age, is clinically characterised by chronic occasional coughing with normal breathing at rest [2, 3]. Diagnostic confirmation requires the documentation of either excess tracheobronchial mucus or abnormal profiles on bronchoalveolar lavage fluid (BALF) cytology [4]. One of the exclusion criteria for IAD is the evidence of systemic signs of infection [3, 4]. Among various extrinsic risk-factors, increased proportions of bacteria (mainly *Streptococcus zooepidemicus* and *Actinobacillus/Pasteurella* spp.) isolated from tracheal wash (TW) have repeatedly been associated with clinical signs of IAD in both young and older Thoroughbred racehorses [5, 6].

The recently revised Consensus Statement on equine IAD pointed out the lack of conclusive evidence of a relationship between viral infections with this syndrome [4]. Experimental inoculations recently performed with either equine rhinitis virus -A (ERAV) or equid herpesvirus −2 (EHV-2) leaded to the observation of respiratory clinical signs and/or abnormal cytological profiles that persisted for up to 21 days after challenge [7, 8]. The potential implication of different viruses in airway inflammation and/or poor racing performance has also been previously investigated in an epidemiological manner. These studies were either based on serological analyses [9–11], or more recently on direct detection of viral genome by PCR in nasal swabs or BALF samples [12, 13].

While a significant association has recently been found between seropositivity to ERAV and diagnosis of IAD [13], the use of antibody titres largely appeared to be of limited value in the clinical context of this syndrome [6, 14]. Positive PCR for EHV-2 in nasal swabs, but not in BALF, was also significantly associated with diagnosis of IAD in a recent case-control study [13]. Conversely, clinical signs of respiratory disease were not associated with either the presence or the level of shedding of EHV-2 in nasal swabs [12]. To date, the use of quantitative PCR on respiratory samples in relation to IAD has been described for a very limited number of viruses only [12]. Furthermore, no epidemiological data on viral loads from tracheal samples are currently available, while previously found to be potentially relevant in experimental conditions [7, 8].

The aims of this study were to determine: 1) the prevalence and incidence of viral genome detection in the respiratory tract of racehorses at training; 2) correlations between viral loads in nasopharyngeal swabs (NS) and TW; and 3) the association between virus detection/quantification and clinical signs of airway inflammation. We hypothesised that respiratory viruses might be significant risk-factors of IAD.

## Methods

### Study design

A cohort of French Standardbred Trotters was investigated on a monthly basis (3 to 5 weeks) over 27 consecutive months (November 2012 – January 2015). Three veterinary practitioners located in Normandy (France) systematically visited a total of 8 different training yards (respectively 3, 3 and 2 each). Among these, 4 yards participated for the whole period, 2 declined after respectively 4 and 10 months and were replaced by 2 others yards . At the time of inclusion, 5 horses per yard were randomly selected among those complying with the inclusion criteria: at least 2 years old; in active training or racing; free of any clinical sign of respiratory disease. Any horse leaving the yard during the longitudinal study was replaced by another one from the same yard, in accordance with the inclusion criteria.

### Sampling and data collection

Horses were examined and sampled either at rest or at least 2 h after any exercise. Venous blood samples were collected for haematological assessment, in order to rule out any systemic disease. Although being clinically healthy at the time of inclusion, some horses eventually became clinically affected throughout the study. Presence of respiratory clinical signs, including nasal discharge and coughing at the time of sampling time was then systematically noticed. Tracheal mucus was systematically scored (grade 1–5), according to the previously published scale [15]. NS were taken from the nasopharynx using tailor-made 40 cm long stems ended with a 3 cm long and 1 cm diameter cotton swab (Coveto, Montaigu, France), and immediately placed into 4 ml of transport medium. TW were collected trans-endoscopically [7], by instilling and re-aspirating 40 ml of sterile isotonic saline, and kept in serum tubes for bacteriology and EDTA tubes for respectively cytology and molecular biology. All samples were kept refrigerated and immediately sent to the laboratory for processing within 24 h after collection.

### Cytology and bacteriology

For each TW, 200 μL of fluid was systematically cytocentrifuged (80 *g*, 10 min; Shandon Cytospin, Waltham, USA) and stained with May-Grünwald-Giemsa. Differential cell counts were performed on 300 leukocytes. Standard quantitative bacterial investigations were also performed on TW [16]. Identification and quantification were performed only on samples exhibiting up to 3 different strains. Samples with isolation counts < 10^4^ colony-forming units (CFU)/ml were considered as negative. Isolated bacteria were classified as either common pathogens or commensals [17].

### Molecular biology

All qPCR have been validated in respiratory samples, based on the NF-U-47600-2 AFNOR norm [18]. Detection of the following viruses have been assessed (Additional file 1): equid herpesvirus (EHV) -1, −4, −2 and −5; equine rhinitis virus -A (ERAV), Equine rhinitis virus -B (ERBV, now erbovirus A); equine adenovirus (EAdV) -1 and −2 (now equine mastadenovirus -A and -B); equine coronavirus (ECoV) and equine influenza virus (EIV).

Respectively 2 ml of NS transport medium and 10 ml of TW were centrifuged (2000 *g*; 15 min; 4 °C), and pellets were re-suspended with 500 μl of supernatant. Extraction of DNA/RNA was made from 140 μl of each sample, using the QIAamp® RNA viral Mini Kit (Qiagen, Courtaboeuf, France) according to the manufacturer’s instructions. Concentrated DNA/RNA was then stored at −80 °C until used. Virus-specific qPCR were performed in a total volume of 25 μl, and included a negative control (distilled water) and serial dilutions of synthetic DNA/RNA (respectively plasmids and transcripts of plasmids for each targeted region). Amplification of the synthetic sequence “myIC” [19] was also performed, to confirm absence of PCR inhibitors in the nucleic acid extracts. As previously described [20], viral loads for each respiratory fluid were expressed without normalisation against a preselected cell number or amount of DNA.

Based on the NF-U-47600-2 AFNOR norm, all respiratory samples have systematically been classified, for each investigated virus, in one of the following groups:

- ‘Negative’, when no characteristic amplification curve was present;
- ‘Positive’ (detection), when the characteristic amplification curve was present.

The ‘positive’ group has also been divided in the 2 following subcategories:

- ‘Non-quantifiable’, when the viral load was < Limit of Quantification (LoQ);
- ‘Quantifiable’ (quantification), when the viral load was ≥ LoQ.

### Statistical analysis

Descriptive statistics were performed for all relevant variables. Both overall prevalence and monthly incidence were determined, and binomial 95% confidence intervals (CI) calculated. Continuous data which were not normally distributed, as assessed by Shapiro-Wilk *W* test, were log 10 transformed to normalise distribution and presented as ‘median (1^st^ – 3^rd^ quartile)’. Correlation between viral loads and measure of agreement for NS and TW were evaluated using Pearson’s coefficient and Cohen’s kappa coefficient (*κ*), respectively. Association between detection/quantification of pathogens and clinical/diagnostic variables for airway inflammation were assessed using χ^2^ test, and calculation of corresponding odds ratio (OR). Data analyses were conducted using Prism 6 (GraphPad, La Jolla, USA) and NCSS 9 (NSS - LLC, Kaysville, USA) software. Values of *P* < 0.05 were considered significant.

## Results

### Study population

**Fig. 1:**
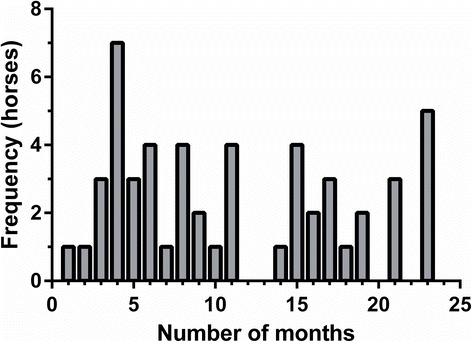
Frequency distribution of number of months contributed to the study (*n* = 52 horses)

A total of 52 horses contributed to the study, and 581 samplings have been conducted over the period. The median number of months in which the same horse has been sampled is 9.5 months (5.0–17.0; Fig. 1). The cohort was composed by 24 geldings, 16 females and 12 males, with a median age of 4.0 (3.0–5.0) years at the time of recruitment.

### Clinical signs and airway inflammation

Clinical signs were recorded for 424/581 (73.0%) samplings. The overall prevalence of coughing and nasal discharge was 6.1% (CI 4.7–7.8) with incidence rates of 4.5 (CI 3.3–6.0) and 5.4 (CI 4.1–7.0) new cases/100 horses/month, respectively. The overall prevalence of excess tracheal mucus (score ≥ 2/5) was 19.1% (CI 16.7–21.7) with an incidence rate of 15.9 new cases/100 horses/month (CI 13.7–18.3). Similarly, the overall prevalence of increased TW neutrophil proportions (>30%) was 54.2% (CI 51.1–57.3) with an incidence rate of 29.1 new cases/100 horses/month (CI 26.3–32.0).

### Isolation and quantification of bacteria in TW

Overall, 411/581 (70.7%) TW samples were negative (<10^4^ CFU/ml), and 18/581 (3.1%) exhibited > 3 different bacterial strains. Common pathogens have been identified in 14/581 (2.4%) TW samples (Table 1), with commensals also concomitantly isolated in 13/14 (92.9%) samples. In total, commensals were found in 136/581 (23.4%) TW (Additional file 2).

**Table 1:** Identification and quantification of common pathogenic bacteria isolated in tracheal washes

Identification	Samples	CFU/ml (range)
*Escherichia coli*	7	1 × 10^4^–4 × 10^5^
*Actinobacillus equullii*	2	2 × 10^4^; 8 × 10^4^
*Streptococcus zooepidemicus*	2	1 × 10^4^; 1 × 10^4^
*Bordetella bronchiseptica*	1	2.2 × 10^5^
*Pasteurella* spp.	1	1 × 10^4^
*Klebsiella pneumoniae*	1	1 × 10^4^

*CFU* colony-forming unit

### Detection of viral genome by qPCR in NS

**Fig. 2:**
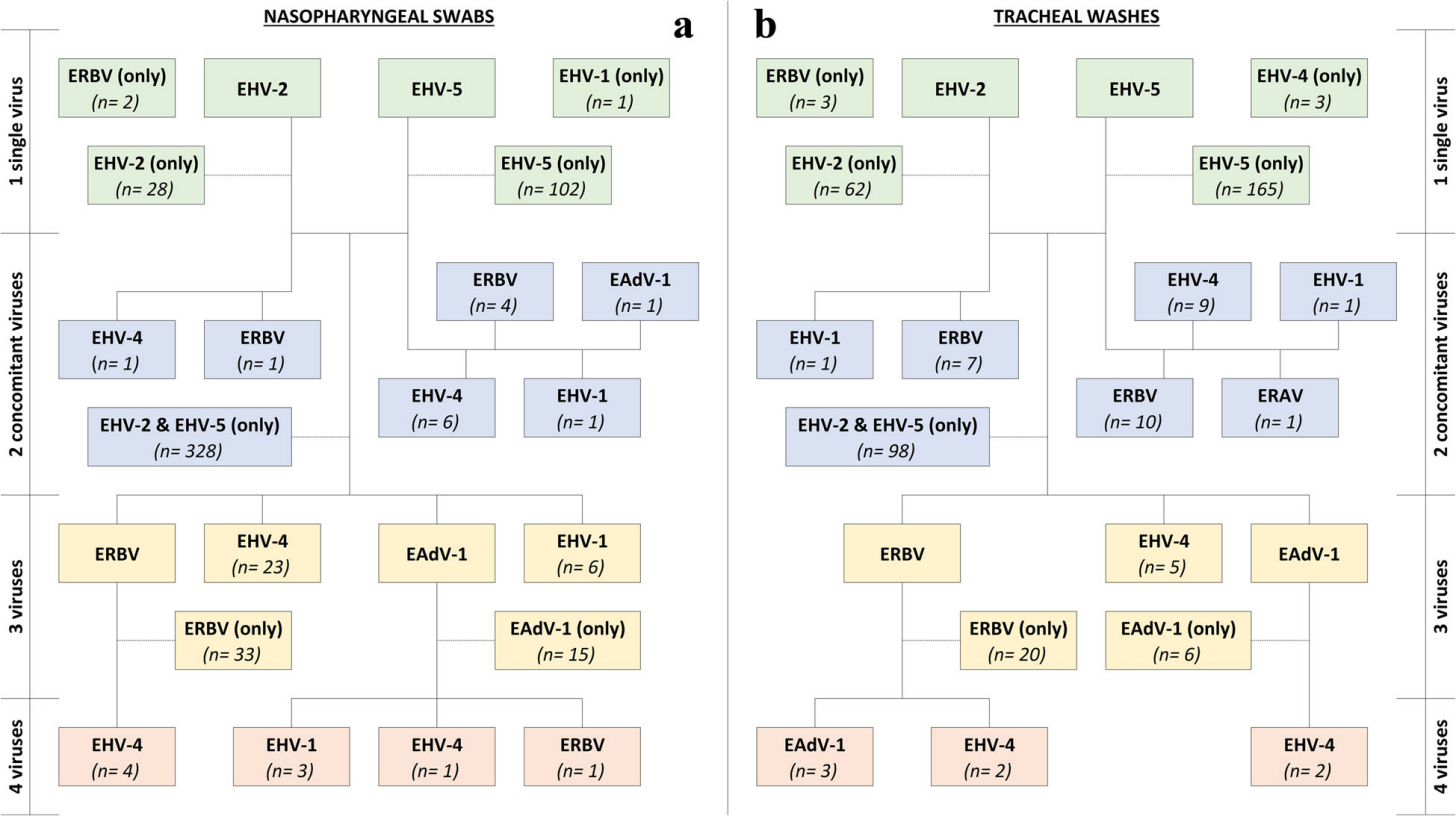
Viral genomes simultaneously detected by qPCR in **a** nasopharyngeal swabs, and **b** tracheal washes. EHV: equid herpesvirus; ERAV: equine rhinitis virus -A; ERBV: equine rhinitis virus -B; EAdV-1: equine adenovirus −1; n: number of ‘positive’ samples

Among the 10 respiratory viruses investigated, 6 have been detected at least once throughout the study; ERAV, EAdV-2, ECoV and EIV have never been detected in any NS. Both γ-herpesviruses (EHV-2 and −5) have been detected in NS at least once for all 52 horses; EHV-4 for 21/52 (40.4%) horses, ERBV for 20/52 (38.5%) horses, EAdV1 for 16/52 (30.8%) horses and EHV-1 for 8/52 (15.4%) horses. In total, 20/581 (3.4%) NS samples were ‘negative’, and at least one virus has been detected in 561/581 (96.6%) NS samples. Among these, genome of one single virus has been detected in 133/561 (23.7%) samples, and DNA/RNA of up to 4 different viruses have been concomitantly detected in the other 428/561 (76.3%) NS (Fig. 2a).

**Fig. 3:**
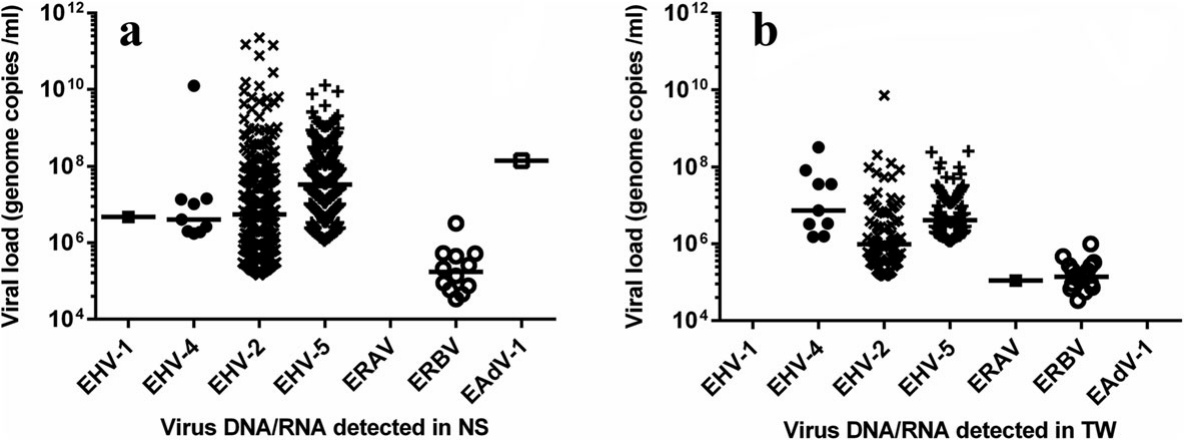
Viral loads for respiratory viruses in **a** ‘quantifiable’ nasopharyngeal swabs, and **b** ‘quantifiable’ tracheal washes. EHV: equid herpesvirus; ERAV: equine rhinitis virus A; ERBV: equine rhinitis virus B; EAdV-1: equine adenovirus −1; horizontal line = median

The overall prevalence of viral genome detection in NS is summarised in Table 2, and respective viral loads detailed in Fig. 3a. Incidences of ‘positive’ NS samples /100 horses/month were respectively 31.5 (CI 28.6–34.5) for EHV-5, 29.4 (CI 26.6–32.3) for EHV-2, 6.0 (CI 4.6–7.7) for ERBV, 5.5 (CI 4.2–7.1) for EHV-4, 2.9 (CI 2.0–4.1) for EAdV-1 and 1.9 (CI 1.1–3.0) for EHV-1. Incidences of ‘quantifiable’ NS samples /100 horses/month were respectively 24.3 (CI 21.7–27.1) for EHV-5, 23.6 (CI 21.0–26.4) for EHV-2, 1.9 (CI 1.1–3.0) for ERBV, 1.5 (CI 0.8–2.5) for EHV-4, 0.2 (CI 0.0–0.7) for EAdV-1 and 0.2 (CI 0.0–0.7) for EHV-1.

**Table 2:** Prevalence of viral genome detection by qPCR in nasopharyngeal swabs and tracheal washes

Virus	Overall prevalence (95% CI)			
	‘Positive’ samples		‘Quantifiable’ samples	
	NS	TW	NS	TW
EHV-5	90.9% (88.9–92.6)	55.4% (52.3–58.5)	58.7% (55.6–61.8)	19.1% (16.7–21.7)
EHV-2	76.4% (73.6–79.0)	35.4% (32.4–38.5)	47.7% (44.6–50.8)	14.6% (12.5–16.9)
ERBV	7.8% (6.2–9.6)	7.8% (6.2–9.6)	2.1% (1.3–3.2)	2.9% (2.0–4.1)
EHV-4	6.0% (4.6–7.7)	3.8% (2.7–5.2)	1.5% (0.8–2.5)	1.5% (0.8–2.5)
EAdV1	3.6% (2.5–4.9)	1.9% (1.1–3.0)	0.2% (0.0–0.7)	neg.
EHV-1	1.9% (1.1–3.0)	0.2% (0.0–0.7)	0.2% (0.0–0.7)	neg.
ERAV	neg.	0.2% (0.0–0.7)	neg.	0.2% (0.0–0.7)
EAdV2	neg.	neg.	neg.	neg.
ECoV	neg.	neg.	neg.	neg.
EIV	neg.	neg.	neg.	neg.

*NS* nasopharyngeal swab, *TW* tracheal wash, *EHV* equid herpesvirus, *ERAV* equine rhinitis virus A, *ERBV* equine rhinitis virus B, *EAdV* equine adenovirus, *ECoV* equine coronavirus, *EIV* equine influenza virus, *neg*. negative

### Detection of viral genome by qPCR in TW

Among the 10 respiratory viruses investigated, 7 have been detected at least once throughout the study; EAdV-2, ECoV and EIV have never been detected in any TW. EHV-5 has been detected in TW at least once for all 52 horses; EHV-2 for 48/52 (92.3%) horses, ERBV for 22/52 (42.3%) horses, EHV-4 for 14/52 (26.9%) horses, EAdV1 for 11/52 (21.2%) horses, EHV-1 and ERAV for respectively 1/52 (1.9%) horses. In total, 183/581 (31.5%) TW were ‘negative’, and at least one virus has been detected in 398/581 (68.5%) TW samples. Among these, genome of one single virus has been detected in 233/398 (58.5%) samples, and DNA/RNA of up to 4 different viruses have been concomitantly detected in the other 165/398 (41.5%) TW (Fig. 2b).

The overall prevalence of viral genome detection in TW is summarised in Table 2, and respective viral loads detailed in Fig. 3b. Incidences of ‘positive’ TW samples /100 horses/month were respectively 27.9 (CI 25.1–30.8) for EHV-5, 24.8 (CI 22.2–27.6) for EHV-2, 7.1 (CI 5.6–8.9) for ERBV, 3.8 (CI 2.7–5.2) for EHV-4, 1.9 (CI 1.1–3.0) for EAdV-1, 0.2 (CI 0.0–0.7) for EHV-1 and 0.2 (CI 0.0–0.7) for ERAV. Incidences of ‘quantifiable’ TW samples /100 horses/month were respectively 14.8 (CI 12.7–17.2) for EHV-5, 12.2 (CI 10.2–14.4) for EHV-2, 2.6 (CI 1.7–3.8) for ERBV, 1.5 (CI 0.8–2.5) for EHV-4 and 0.2 (CI 0.0–0.7) for ERAV.

### NS vs. TW for genome detection and viral loads

Very poor agreements only, according to *κ* coefficients, were found between paired samples (NS and TW) for the detection of each viral genome by qPCR (‘positive’ *vs.* ‘negative’; [Additional file 3]). When concerning ‘quantifiable’ samples only, no significant correlation was found among viral loads in paired samples (NS and TW) for any of the investigated viruses.

### Pathogens vs. clinical signs and airway inflammation

Detection of EHV-2 DNA in TW was significantly associated with coughing (OR 3.1; 95% CI: 1.4–7.1; *P* = 0.01), as were both detection (OR 5.3; CI 2.1–14.0) and quantification (OR 15.0; CI 3.8–60.1) of ERBV RNA in TW (*P* < 0.001). Isolation of commensal bacteria in TW was also significantly associated with coughing (OR 2.7; CI 1.20–6.2; *P* = 0.02). There was no association between any virus or bacteria in TW and nasal discharge. Similarly, there was no significant association between either viral genome detection or quantification in NS and any clinical sign of respiratory disease.

Quantification of at least one virus in TW (‘quantifiable’ sample) was significantly associated with TW neutrophil counts > 30% (OR 1.5; CI 1.1–2.2; *P* = 0.03). Detection of EHV-2 DNA in TW was significantly associated with excess tracheal mucus (OR 2.1; CI 1.2–3.8; *P* = 0.02). Viral loads for EHV-2 in TW were also fairly correlated with neutrophil proportions (*R* = 0.32; CI 0.12–0.51; *P* = 0.003). Conversely, isolation of commensal bacteria was negatively associated with TW neutrophil counts > 30% (OR 0.6; CI 0.4–0.9; *P* = 0.02). There was no association between tracheal mucus score ≥ 2/5 and any other virus or bacteria in TW, or any viral genome detection/quantification in NS.

## Discussion

This study provided incidence and prevalence of viral genome detection by qPCR and corresponding viral loads in both NS and TW of horses at training. In total, 7 of the 10 viruses investigated have been detected at least once in respiratory samples. Along with EAdV-2 and ECoV, EIV genome has never been found in NS or TW throughout the study. This latter was not unexpected since all horses were in active training and racing, and thus vaccinated (every 6 months) at the time of sampling. Alternatively, up to 4 different viral genomes have been concomitantly detected in either NS or TW. Previous studies documented concurrent infections between ERBV and either EHV-4 [21], γ-herpesviruses [21, 22], or EIV [23]. To our knowledge, the present study is the first to report concomitant genome detection of EAdV-1 along with ERBV, EHV-1 or EHV-4.

Among other bacteria, a relatively low prevalence was found for isolation of *Streptococcus zooepidemicus* in TW, when compared with previous longitudinal studies [10, 11]. However, cut-off values of 10^4^ CFU/ml have been used for discriminating negative and positive samples, as described previously [24]. Similarly, identification and quantification have also been performed only on samples with up to 3 different strains. A moderate even significant association between identification of commensals, but not *Streptococcus zooepidemicus*, was significantly associated with coughing in the present study. This unexpected finding suggests that high counts of commensal bacteria in the trachea of athletic horses might opportunistically become pathogenic, along with other viral or environmental challenges. Conversely, coughing has been associated with TW isolation of *Streptococcus zooepidemicus* or *Pasteurellaceae* spp. in a previous case-control study [9]; the odds of airway inflammation being also significantly increased along with CFU/ml [5, 6, 24].

Genome detection for γ-herpesviruses (EHV-2, EHV-5) was highly prevalent in the present longitudinal study, which is in accordance with previously published data [12, 25]. Despite a moderately lower monthly incidence, both EHV-2 and EHV-5 have been detected on at least one occasion for all 52 horses of the study. Due to their capability of viral latency, it might then be determined that each of these horses was a permanent carrier for both EHV-2 and EHV-5. Previously published data also revealed that multiple strains of EHV-2 might be concomitantly detected in nasal swabs or TW of a single horse [7, 26]. However, no phylogeny has been performed for further characterising the strains detected among time in the present study.

Detection of EHV-2 DNA in TW has been associated with coughing and excess tracheal mucus, and viral loads significantly correlated to TW neutrophil proportions. These data confirm previous cytological findings from experimentally induced EHV-2 reactivation [7]; and are also coherent with a recent case-control study in which nasal shedding of EHV-2 was significantly associated with diagnosis of IAD [13]. Based on the growing evidence of its implication in lower airway inflammation; elucidating the underlying EHV-2 pathogenesis warrants further investigation. On the other hand, EHV-5 detection, quantification or viral loads in either NS or TW have not been associated with coughing, nasal discharge nor airway inflammation in this study. Such observation might however not be surprising, since EHV-5 has repeatedly been detected in asymptomatic horses [25, 27]. Alternatively, the clinical signs frequently reported for equine multinodular pulmonary fibrosis, an EHV-5 associated chronic disease, include intermittent fever, weight loss and respiratory distress [28].

Genome detection for ERBV in TW has been significantly associated with coughing in this study, with an increasing OR when higher viral loads were considered. The prevalence of ERBV RNA quantification presently found in NS was slightly higher than previous data [12], which might be partially explained by the differences in sampling methodologies (nasal *vs.* nasopharyngeal swabs) and qPCR assays. Conversely, the monthly incidence of ‘quantifiable’ samples for ERBV was comparable to previously reported serological results, based on complement fixation tests [11]. It has however been recently found that detection of ERBV RNA by PCR in nasal swabs might not coincide with seroconversion [14]. Besides, the low prevalence of ERAV RNA detection in NS or TW was somehow unexpected, since being previously associated with acute clinical cases of respiratory diseases [29]. In addition, a high seroprevalence has been previously described worldwide for this virus [30] and vaccination is currently not available in Europe.

In accordance with results from a previous longitudinal study [14], no significant association was found between any viral genome detection/quantification in NS and clinical signs, excess tracheal mucus or increased TW neutrophil proportions. Interestingly, neither agreement nor correlation has been found between paired samples (NS and TW), for genome detection or viral loads of any investigated virus. In terms of subclinical infectious diseases, findings from upper airways (NS) cannot be considered as representative of lower airways (TW) in the same horse. Moreover, viral genome detection in NS seems not to be relevant in the clinical context of IAD, unlike acute infectious airway troubles [22, 29]. It might then be recommended to perform qPCR in TW samples for any virus detection on horses at training, as previously suggested for a limited number of herpesviruses [31].

One limitation of the present study would be the lack of virus isolation or serological assays in addition to the qPCR performed in respiratory fluids. The demonstration of increased antibody titres however requires two consecutive samplings with a 3 to 4-week interval, which is irrelevant on the field for any concomitant diagnosis of IAD. Apart from viruses and bacteria, non-infectious agents are also likely to be relevant to the development of IAD in horses [4]. Another limitation would then be the absence of environmental investigations within the different training yards throughout the study. Ultra-fine particles (< 10 μm) within the breathing zone of the horse were indeed recently found to be significant risk-factors for excess mucus and increased TW neutrophil proportions [32].

## Conclusions

Detection of ERBV RNA by qPCR in airway samples was prevalent in the present study, and also significantly associated with occurrence of coughing in racehorses at training. Sampling NS only is not sufficient for characterising the clinical relevance of any viral genome detection or quantification. Systematically performing qPCR in TW samples should be recommended, along with other clinical and laboratory investigations, in order to identify any viral component potentially associated with IAD.

## Additional files

Additional file 1: Characteristics of q(RT)-PCR. LoQ: Limit of quantification; EHV: equine herpesvirus; ERAV: equine rhinitis virus A; ERBV: equine rhinitis virus B; EAdV: equine adenovirus; ECoV: equine coronavirus; EIV: equine influenza virus. (DOCX 22 kb)
Additional file 2: Identification and quantification of major commensals (isolated in > 10% positive tracheal washes). CFU: colony-forming unit. (DOCX 15 kb)
Additional file 3: Agreement between paired nasopharyngeal swabs and tracheal washes for the detection of viral genome by qPCR. EHV: equid herpesvirus; ERBV: equine rhinitis virus B; EAdV-1: equine adenovirus −1; n.a.: not applicable. Divergent = ’positive’ in one sample (TW or NS) and ‘negative’ in the corresponding paired sample. (DOCX 15 kb)

## Abbreviations

BALF: Bronchoalveolar lavage fluid
CFU: Colony-forming unit
CI: Confidence interval
EAdV: Equine adenovirus
ECoV: Equine coronavirus
EHV: Equid herpesvirus
EIV: Equine influenza virus
ERAV: Equine rhinitis virus -A
ERBV: Equine rhinitis virus -B
IAD: Inflammatory airway disease
LoQ: Limit of quantification
NS: Nasopharyngeal swab
TW: Tracheal wash
