# Supplementary material for: Multiple molecular detection of respiratory viruses and associated signs of airway inflammation in racehorses
Source: Virol J. 2016 Nov 29;13:197. doi: 10.1186/s12985-016-0657-5 (PMC5129218; doi:10.1186/s12985-016-0657-5)
Supplement: Additional file 1: — Characteristics of q(RT)-PCR. LoQ: Limit of quantification; EHV: equine herpesvirus; ERAV: equine rhinitis virus A; ERBV: equine rhinitis virus B; EAdV: equine adenovirus; ECoV: equine coronavirus; EIV: equine influenza virus. (DOCX 22 kb) [file 12985_2016_657_MOESM1_ESM.docx]

**Additional file 1. Characteristics of q(RT)-PCR.**

| **Assay** | **Target gene** | **Primer and probe sequences (5’-3’)** | **Product size (bp)** | **Amplification** | | **Ref.** | **LoQ (genome copies /ml)** |
| --- | --- | --- | --- | --- | --- | --- | --- |
| **myIC** | myIC | F : CTAACCTTCGTGATGAGCAATCG  R : GATCAGCTACGTGAGGTCCTAC  P : **YY**-AGCTAGTCGATGCACTCCAGTCCTCCT-**BHQ1** | 144 | 50 °C 2 min 95 °C 10 min  95 °C 15 s │ 60 °C 1 min │ | x40 | (Deer et al., 2010) | / |
| **EHV-1** | gB | F : CATGTCAACGCACTCCCA  R : GGGTCGGGCGTTTCTGT  P : **FAM**-CCCTACGCTGCTCC-**MGB-NFQ** | 63 | 95 °C 10 min 95 °C 15 s │ 60 °C 1 min │ | x45 | (Diallo et al., 2006) | 72 000 |
| **EHV-4** | gB | F : GGGCTATTGGATTACAGCGAGAT  R : TAGAATCGGAGGGCGTGAAG  P : **VIC**-CAGCGCCGTAACCAG-**MGB-NFQ** | 58 | 95 °C 10 min 95 °C 15 s │ 60 °C 1 min │ | x45 | (Diallo et al., 2007) | 43 200 |
| **EHV-2** | gB | F : CCTCAACCTGACTGACATACCCA  R : TTTAGAATAGAGCTCAATCACCTTAAAATC  P : **FAM**-CACCATGATTACCCTGAACCTGTCTCTGGT-**BHQ1** | 100 | 95 °C 10 min 95 °C 15 s │ 60 °C 1 min │ | x45 | (Brault et al., 2010) | 1 040 |
| **EHV-5** | gB | F : AACCCGCCGTGCATCA  R : AGGCGCCACACACCCTAA  P : **FAM**-ACAACACCACCAACCCCTTTCTGCTG-**BHQ1** | 66 | 95 °C 10 min 95 °C 15 s │ 60 °C 1 min │ | x45 | (Hue et al., 2014) | 2 000 |
| **EAdV1** | Hexon | F : GATGCTTCCACAATGGTCCT  R : CTCGGTGGTGACATCGTG  P : **FAM**-GAATACCTGTCGCCCGCTC-**BHQ1** | 163 | 50 °C 2 min 95 °C 10 min  95 °C 15 s │ 55 °C 1 min │ | x40 | Present study | 5 500 |
| **EAdV2** | Hexon | F : gaacgtaccaccgaaaggtt  R : gtcccgcggatgtaaaagta  P : **FAM**-cggtttcagttggccgttgg-**BHQ1** | 158 | 50 °C 2 min 95 °C 10 min  95 °C 15 s │ 55 °C 1 min │ | x40 | Present study | 8 300 |
| **ERAV** | 5’UTR | F : CCAGGTAACCGGACAGCG  R : GGCAGCGCTACCACAGG  P : **FAM**-CATTGCTCTGGATGGTGT**-MGB-NFQ** | 118 | 42 °C 5 min 95 °C 10 s  95 °C 5 s │ 56 °C 30 s │ | x40 | (Quinlivan et al., 2010) | 80 |
| **ERBV** | 5’UTR | F : TGATGCTTGGCTCTCAGAAA  R : GCAAACGACCAACACATCAA  P : **FAM**-CTTCCAACTAAACCC**-MGB-NFQ** | 132 | 42 °C 5 min 95 °C 10 s  95 °C 5 s │ 56 °C 30 s │ | x40 | (Quinlivan et al., 2010) | 240 |
| **ECoV** | M | F : GGTGGAGTTTCAACCCAGAA  R : AGGTGCGACACCTTAGCAAC  P : **FAM-**CCACAATAATACGTGGCCACCTTTA-**BHQ1** | 204 | 42 °C 5 min 95 °C 10 s  95 °C 5 s │  60 °C 30 s │ | x40 | (Miszczak et al., 2014b) | 100 000 |
| **EIV** | M | F : AGATGAGYCTTCTAACCGAGGTCG  R : TGCAAANACATCYTCAAGTCTCTG  P : **FAM**-TCAGGCCCCCTCAAAGCCGA-**BHQ1** | 102 | 50 °C 20 min 95 °C 5 min  95 °C 15 s │ 58 °C 45 s │ | x40 | (Heine et al., 2007) | 1 120 |

LoQ: Limit of quantification; EHV: equine herpesvirus; ERAV: equine rhinitis virus A; ERBV: equine rhinitis virus B; EAdV: equine adenovirus; ECoV: equine coronavirus; EIV: equine influenza virus.
