# Supplementary material for: Multiple molecular detection of respiratory viruses and associated signs of airway inflammation in racehorses
Source: Virol J. 2016 Nov 29;13:197. doi: 10.1186/s12985-016-0657-5 (PMC5129218; doi:10.1186/s12985-016-0657-5)
Supplement: Additional file 2: — Identification and quantification of major commensals (isolated in > 10% positive tracheal washes). CFU: colony-forming unit. (DOCX 15 kb) [file 12985_2016_657_MOESM2_ESM.docx]

**Additional file 2. Identification and quantification of major commensals (isolated in > 10% positive tracheal washes).**

| Identification | Samples | CFU/ml (range) |
| --- | --- | --- |
| *Pantoea* spp | 27 | 1 x10^4^ – 6 x10^6^ |
| *Pseudomonas* spp | 25 | 1 x10^4^ – 4 x10^7^ |
| *Micrococcus* spp | 24 | 1 x10^4^ – 9 x10^5^ |
| Coagulase negative  *Staphylococcus* | 21 | 1 x10^4^ – 4 x10^6^ |
| Alpha-hemolytic *Streptococcus* | 16 | 1 x10^4^ – 8 x10^6^ |

CFU: colony-forming unit.
