# Supplementary material for: Multiple molecular detection of respiratory viruses and associated signs of airway inflammation in racehorses
Source: Virol J. 2016 Nov 29;13:197. doi: 10.1186/s12985-016-0657-5 (PMC5129218; doi:10.1186/s12985-016-0657-5)
Supplement: Additional file 3: — Agreement between paired nasopharyngeal swabs and tracheal washes for the detection of viral genome by qPCR. EHV: equid herpesvirus; ERBV: equine rhinitis virus B; EAdV-1: equine adenovirus −1; n.a.: not applicable. Divergent = ’positive’ in one sample (TW or NS) and ‘negative’ in the corresponding paired sample. (DOCX 15 kb) [file 12985_2016_657_MOESM3_ESM.docx]

**Additional file 3. Agreement between paired nasopharyngeal swabs and tracheal washes for the detection of viral genome by qPCR.**

| Virus | ‘Negative’  Samples | ‘Positive’  samples | Divergent  samples | *κ* (95% IC) |
| --- | --- | --- | --- | --- |
| EHV-5 | 33 | 548 | 246 | 0.07 (0.03 – 0.12) |
| EHV-2 | 102 | 479 | 308 | 0.08 (0.03 – 0.14) |
| ERBV | 496 | 85 | 80 | 0.04 (-0.06 – 0.13) |
| EHV-4 | 531 | 50 | 43 | 0.21 (0.06 – 0.36) |
| EAdV-1 | 551 | 29 | 27 | 0.11 (-0.05 – 0.27) |
| EHV-1 | 569 | 12 | 12 | n.a. |

EHV: equid herpesvirus; ERBV: equine rhinitis virus B; EAdV-1: equine adenovirus -1; n.a.: not applicable. Divergent = ‘positive’ in one sample (TW or NS) and ‘negative’ in the corresponding paired sample.
